# Multimodal Neurologic Monitoring in Patients Undergoing Extracorporeal Membrane Oxygenation

**DOI:** 10.7759/cureus.59476

**Published:** 2024-05-01

**Authors:** Khwaja Siddiqui, Muhammad U Hafeez, Ali Ahmad, Syed O Kazmi, Subhasis Chatterjee, Eric Bershad, Mohammad Hirzallah, Chethan Rao, Rahul Damani

**Affiliations:** 1 Neurology, Baylor College of Medicine, Houston, USA; 2 Neurology, University of Texas Medical Branch, Galveston, USA; 3 Neurology, Salem Health Hospitals & Clinics, Salem, USA; 4 Cardiovascular Surgery, The Texas Heart Institute, Houston, USA; 5 Neurocritical Care, Baylor College of Medicine, Houston, USA

**Keywords:** transcranial doppler sonography, electroencephalography, intracranial hemorrhage, stroke, neurophysiological monitoring, extracorporeal membrane oxygenation

## Abstract

Introduction

Extracorporeal membrane oxygenation (ECMO) is associated with a high rate of neurologic complications. Multimodal neurologic monitoring (MNM) has the potential for early detection and intervention. We examined the safety and feasibility of noninvasive MNM during ECMO. We hypothesized that survivors and non-survivors would have meaningful differences in transcranial Doppler (TCD) sonography and electroencephalographic (EEG) characteristics, which we aimed to identify. We also investigated adverse neurologic events and attempted to identify differences in EEG and TCD characteristics among patients based on the type of ECMO and the occurrence of these events.

Material and methods

We performed an observational study on all patients undergoing ECMO at Baylor St. Luke's Medical Center’s critical care unit in Houston, Texas, United States, from January 2017 to February 2019. All patients underwent a noninvasive MNM protocol.

Results

NM was completed in 75% of patients; all patients received at least one component of the monitoring protocol. No adverse events were noted, showing the feasibility and safety of the protocol. The 60.4% of patients who did not survive tended to be older, had lower ejection fractions, and had lower median right middle cerebral artery (MCA) pulsatility and resistivity indexes. Patients undergoing venoarterial (VA)-ECMO had lower median left and right MCA velocities and lower right Lindegaard ratios than patients who underwent venovenous-ECMO. In VA-ECMO patients, EEG less often showed sleep architecture, while other findings were similar between groups. Adverse neurologic events occurred in 24.7% of patients, all undergoing VA-ECMO. Acute ischemic stroke occurred in 22% of patients, intraparenchymal hemorrhage in 4.9%, hypoxic-ischemic encephalopathy in 3.7%, subarachnoid hemorrhage in 2.5%, and subdural hematoma in 1.2%.

Conclusion

Our results suggest that MNM is safe and feasible for patients undergoing ECMO. Certain EEG and TCD findings could aid in the early detection of neurologic deterioration. MNM may not just be used in monitoring patients undergoing ECMO but also in prognostication and aiding clinical decision-making.

## Introduction

Extracorporeal membrane oxygenation (ECMO) provides mechanical circulatory support for patients with life-threatening cardiogenic shock, decompensated heart failure, or respiratory failure. It can be used as temporary support to await organ recovery or as a bridge to durable mechanical circulatory support or transplantation [1]. ECMO can be performed in a venovenous (VV-ECMO) or venoarterial (VA-ECMO) configuration. VV-ECMO provides oxygenation and ventilation in respiratory failure; VA-ECMO provides circulatory support while allowing for ventricular recovery, if possible, in cardiogenic shock [2].

While ECMO can be a lifesaving treatment, it also has high rates of complications and neurologic adverse events. Registry analyses from the Extracorporeal Life Support Organization (ELSO) revealed that the incidences of neurologic adverse events and mortality were 15% and 89% during VA-ECMO and 7% and 75-80% during VV-ECMO [3,4]. Neurologic adverse events associated with ECMO include ischemic and hemorrhagic strokes and seizures [5].

Furthermore, ECMO cannulation creates unique challenges for neurologic monitoring [6]. Neurologic monitoring of patients on ECMO can be confounded by ongoing sedation and by medical comorbidities, including acute organ dysfunction; as a result, neurologic changes may go unnoticed until they lead to significant adverse events.

The optimal method of monitoring the neurologic status or predicting prognosis in ECMO-supported patients has not been established. Hence, there has been an interest in noninvasive multimodal neurologic monitoring (MNM) of these patients. Thus far, there is no consensus protocol, and the utility and feasibility of this approach have yet to be determined. A variety of techniques have been reported, including various combinations of frequent neurologic exams, transcranial Doppler (TCD) sonography [7], electroencephalography (EEG), near-infrared spectroscopy, and somatosensory evoked potentials [8]. The majority of published studies of MNM pertain to neonatal and pediatric patients; few have examined its use in adult patients.

The primary objective of our study was to determine the safety and feasibility of noninvasive MNM in ECMO-supported patients. We hypothesized that non-survivors would have altered cerebral autoregulation that could lead to higher TCD velocities, resistivity index (RI), and pulsatility index (PI), along with EEG changes such as loss of normal electrographic findings, epileptiform discharges or seizures, and focal asymmetry. Hence, the secondary objective of our study was to identify these differences. We also sought to investigate the incidence of adverse neurologic events and identify further differences in EEG and TCD characteristics among patients according to the type of ECMO they underwent and the occurrence of neurologic adverse events.

## Materials and methods

Study design and patient selection

We conducted a retrospective analysis of prospectively collected data from patients who underwent noninvasive MNM while on ECMO at Baylor St. Luke's Medical Center, Houston, Texas, United States, from January 2017 to February 2019. The project was a collaborative quality improvement initiative between the cardiovascular ICU and neurologic ICU services. Data were obtained from an institutional review board (IRB)-approved, prospectively maintained ECMO database (BCM IRB (H-43621)), as well as from the patient’s electronic medical record, and de-identified. Because of the study’s retrospective design, the IRB waived the consent requirement. Using chart review, we followed 94 patients for up to one year after discharge, with no loss to follow-up.

Patient care and monitoring

The Quadrox oxygenator and Rotaflow pump (Getinge Group, Gothenburg, Sweden) were used to provide ECMO support. Most VV-ECMO patients were cannulated with a bicaval dual-lumen catheter (Avalon Elite; Getinge Group). Most VA-ECMO patients were cannulated peripherally in a bifemoral configuration. A multidisciplinary team that included a cardiovascular ICU attending physician, cardiologists, cardiac surgeons, perfusionists, nurses, pharmacists, and respiratory therapists rounded twice daily on all ECMO patients.

All patients were monitored with an arterial catheter and a central venous catheter; many patients also had a pulmonary artery catheter. Sedation during mechanical ventilation was maintained with a combination of sedatives (propofol and dexmedetomidine) and analgesics (fentanyl and hydromorphone) titrated to a Richmond Agitation Sedation Score of −1 unless clinical circumstances necessitated modification. Cardiac function was routinely assessed with transthoracic echocardiography. The mean arterial pressure was targeted at greater than 65-70 mmHg with a combination of volume resuscitation and vasopressors with or without inotropes, or by adjusting ECMO flow rates. Blood flow, fraction of inspired oxygen (FiO2), and ECMO gas flow were titrated to maintain a partial pressure of carbon dioxide (PCO2) in the 35-45 mmHg range and a partial pressure of oxygen (PO2) in the 60-150 mmHg range. Unfractionated heparin was used for anticoagulation and was monitored with activated partial thromboplastin time and thromboelastography. As needed, routine blood glucose monitoring and insulin infusion were used to maintain a glucose level of 110-180 mg/dL [9].

MNM protocol

The multimodal monitoring protocol included a combination of portable head CT, EEG, and TCD, along with frequent neurologic examinations while the patient was off sedation. When ECMO was initiated, the cardiovascular ICU team would contact the neurologic ICU team to start MNM. All patients had a baseline neurologic exam off sedation as soon as they were hemodynamically stable. This was followed by daily neurological assessments, which were done off sedation when possible by a neurointensivist for the first three days and extended based on the clinical course. Patients also underwent continuous video EEG monitoring for the first 24 hours, or longer if clinically indicated. Daily TCDs were also performed for three consecutive days whenever feasible. When possible, head CT was performed on days 1 and 3 after ECMO initiation and repeated subsequently on an individual basis if clinically indicated.

EEG was performed using a standard bipolar montage on a Nihon Kohden machine according to the 10-20 configuration and was interpreted by a certified neurophysiologist. The following data were collected retrospectively from the EEG report: epileptiform discharges, electrographic seizures, EEG variability, EEG reactivity, focal asymmetry, presence and pattern of the posterior dominant rhythm, presence of generalized periodic discharges, triphasic morphology, sharp waves, periods of discontinuity, and sleep architecture.

TCD studies were performed by an experienced technician. Variables that were collected included middle cerebral artery (MCA) velocities, internal carotid artery (ICA) velocities, Lindegaard ratios (LRs), and MCA PI and RI. The median of each of these variables for each patient was used for the analysis.

Subgroup analysis

A comparative analysis was performed after dichotomizing patients by survival and type of ECMO. Patients were categorized as survivors or non-survivors. Non-survivors were defined as patients who had died before discharge, and survivors were defined as patients who had any other disposition. Neurologic adverse events were categorized as being present or absent. A neurologic adverse event was identified by imaging and included evidence of a new finding of acute ischemic stroke, intraparenchymal hemorrhage, subarachnoid hemorrhage, subdural hemorrhage, or hypoxic-ischemic encephalopathy. The type of ECMO was defined as either VA-ECMO or VV-ECMO; patients who received both types were categorized in the VA-ECMO group.

Statistical analysis

Categorical variables were expressed as absolute numbers and percentages. The Fisher’s exact test and the chi-square test were used, depending on the number of observations. Continuous variables were analyzed with a histogram to check for skew and kurtosis. Normally distributed variables were analyzed with the independent samples t-test and expressed as means and standard deviations, while non-normally distributed variables were analyzed with the Mann-Whitney U test and expressed as medians and quartiles. Binary logistic regression could not be performed because of the small sample size. A p-value < 0.05 was considered statistically significant. All statistical analyses were carried out with IBM SPSS Statistics for Windows, Version 21.0 (Released 2012; IBM Corp., Armonk, NY, USA).

## Results

A total of 94 patients underwent ECMO from January 2017 to February 2019: nine (9.6%) underwent VV-ECMO, and 85 (90.4%) underwent either VA-ECMO alone (n = 72, 76.6%) or a combination of VA- and VV-ECMO (n = 13, 13.8%). TCD was performed in 75 (79.8%) patients, and EEG was performed in 74 (78.7%) patients. At least one head CT was performed in 89 (94.6%) patients: 56 (59.6%) patients had two scans, and 23 (24.4%) patients had more than two scans. More than half of the CT scans were completed within 24 hours of the start of ECMO. The most common reason for delay in obtaining a CT scan was hemodynamic instability. No adverse events were documented due to MNM in these patients.

Of the 91 patients for whom survival data were available, 55 (60.4%) did not survive (62.2%) in the VA-ECMO group and 4 (44.4%) in the VV-ECMO group. Non-survivors were more likely to be older (mean age 60.9 vs. 53.3 y, p = 0.006) and to have an ejection fraction less than 35% (54.5% vs. 33.3%, p = 0.05). They also had a lower median right MCA PI (0.93 vs. 1.09, p = 0.021) and RI (0.59 vs. 0.63, p = 0.04). Non-survivors had shorter hospital lengths of stay (15 vs. 34 days, p < 0.001) but had longer ECMO courses (8 vs. 6 days, p = 0.004) and were more likely to have an acute ischemic stroke (26.4% vs. 8.6%, p = 0.04) (Tables 1, 2).

**Table 1:** Comparison of baseline characteristics between ECMO survivors and non-survivors A p-value <0.05 was considered statistically significant. ARDS, acute respiratory distress syndrome; ECMO, extracorporeal membrane oxygenation; EF, ejection fraction

	Non-survivors (n = 55)	Survivors (n = 36)	p-value
Age, mean (SD), y	60.87 (12.04)	53.31 (13.33)	0.006
Indication for ECMO, No. (%)			
Respiratory failure	20 (36.4%)	10 (27.8%)	0.5
Cardiogenic shock or cardiac arrest	34 (61.8%)	24 (66.7%)	0.66
Septic shock	3 (5.5%)	4 (11.1%)	0.43
Cardiac surgery	8 (14.5%)	5 (13.9%)	0.93
Gender, No. (%)			
Male	33 (60%)	26 (72.2%)	0.23
Female	22 (40%)	10 (27.8%)	
Race, No. (%)			0.93
Caucasian	31 (62%)	20 (58.8%)	
African American	10 (20%)	8 (23.5%)	
Other	9 (18%)	6 (17.6%)	
Medical history, No. (%)			
Sepsis	27 (49.1%)	12 (33.3%)	0.14
ARDS	19 (34.5%)	9 (25%)	0.36
Lung transplant	6 (10.9%)	3 (8.3%)	0.99
Heart failure	35 (64.8%)	21 (58.3%)	0.53
Atrial fibrillation	19 (34.5%)	18 (51.4%)	0.13
Coronary artery disease	29 (52.7%)	18 (50%)	0.8
Myocardial infarction	9 (16.4%)	8 (22.2%)	0.59
Coronary artery bypass grafting	12 (22.2%)	8 (22.2%)	0.99
Length of stay, median (IQR), d	15 (9, 30)	34 (23.25, 59.50)	<0.001
ECMO duration, median (IQR), d	8 (6, 13)	6 (4, 8)	0.004
Use of mechanical circulatory support, No. (%)	22 (40%)	19 (52.8%)	0.28
Type of ECMO, No. (%)			0.47
VA-ECMO	51 (62.2%)	31 (37.8%)	
VV-ECMO	4 (44.4%)	5 (55.6%)	
The lowest recorded EF <35%, No. (%)	30 (54.5%)	12 (33.3%)	0.05
Wall motion abnormality, No. (%)	27 (49.1%)	18 (50%)	0.93
Left atrial enlargement, No. (%)	16 (29.1%)	9 (25%)	0.67
Life-threatening arrhythmia, No. (%)	20 (36.4%)	11 (30.6%)	0.57

**Table 2:** Comparison of neurological outcomes between ECMO survivors and non-survivors A p-value <0.05 was considered statistically significant. ^a^ Data missing for 24 patients ECMO, extracorporeal membrane oxygenation; EEG, electroencephalography; GPD, generalized periodic discharge; ICA, internal carotid artery; L MCA, left middle cerebral artery; LR, Lindegaard ratio; PDR, posterior dominant rhythm; PI, pulsatility index; R MCA, right middle cerebral artery; TCD, transcranial Doppler

	Non-survivors (n = 55)	Survivors (n = 36)	p-value
Head CT findings, No. (%)			
Hypoxic-ischemic encephalopathy	2 (3.8%)	1 (2.9%)	0.99
Acute ischemic stroke	14 (26.4%)	3 (8.6%)	0.04
Intracranial hemorrhage	4 (7.7%)	0 (0%)	0.15
Subarachnoid hemorrhage	1 (1.9%)	1 (2.9%)	0.99
Subdural hemorrhage	1 (1.9%)	0 (0%)	0.99
TCD variables, median (IQR) (n = 75)^a^			
Average L MCA velocity	43.86 (56.8, 68.46)	59.3 (46.89, 73.13)	0.49
Average L ICA velocity	38.5 (31.31, 46.27)	40.2 (35.2, 45.4)	0.76
Average L LR	1.51 (1.15, 1.90)	1.53 (1.21, 1.77)	0.99
Average R MCA velocity	48.3 (40.7, 72.1)	55.03 (45.06, 72.49)	0.36
Average R ICA velocity	35.05 (28.55, 47.74)	37.93 (30.09, 44.49)	0.53
Average R LR	1.54 (1.26, 2.06)	1.43 (1.19, 2.08)	0.8
Average L MCA PI	0.94 (0.69, 1.36)	1.10 (0.82, 1.38)	0.16
Average R MCA PI	0.93 (0.54, 1.22)	1.09 (0.94, 1.31)	0.021
Average L MCA reactivity index	0.57 (0.45, 0.70)	0.64 (0.55, 0.72)	0.09
Average R MCA reactivity index	0.59 (0.41, 0.68)	0.63 (0.58, 0.71)	0.04
EEG findings, No. (%)			
Clinical seizure	1 (1.8%)	0 (0%)	0.99
Epileptiform discharges	2 (4.4%)	0 (0%)	0.53
Electrographic seizure	1 (2.2%)	0 (0%)	0.99
EEG variability	20 (48.8%)	12 (48%)	0.99
EEG reactivity	19 (44.2%)	11 (47.8%)	0.77
Focal asymmetry	5 (10.9%)	1 (4%)	0.41
PDR	3 (6.5%)	5 (20.8%)	0.11
GPDs with triphasic morphology	6 (13%)	4 (16%)	0.73
Sharp waves	5 (10.9%)	1 (4%)	0.41
Periods of discontinuity	1 (2.2%)	2 (8%)	0.28
Sleep architecture	2 (4.3%)	2 (8%)	0.61

VA-ECMO patients tended to be older (59.1% vs. 45.9%, p = 0.004) and were more likely to present with cardiogenic shock or cardiac arrest as an indication for ECMO (69.4% vs. 11.1%, p < 0.001) and to have a history of heart failure (69.0% vs. 0%, p < 0.001) and coronary artery disease (55.3% vs. 11.1%, p = 0.01). Furthermore, VA-ECMO patients were more likely to have an ejection fraction lower than 35% (46.8% vs. 0%, p = 0.03), wall motion abnormalities (55.3% vs. 0%, p = 0.003), life-threatening arrhythmias (38.3% vs. 0%, p = 0.024), and use of mechanical circulatory support (50.6% vs. 0%, p = 0.003). VA-ECMO patients were less likely to have respiratory failure (28.2% vs. 77.8%, p = 0.005) or acute respiratory distress syndrome (23.5% vs. 100%, p < 0.001) as an indication for ECMO.

In terms of TCD variables, patients undergoing VA-ECMO had lower median left MCA (56.8% vs. 78.7%, p = 0.027) and right MCA velocities (49.7% vs. 75.0%, p = 0.026), along with lower right LR (1.4 vs. 2.2, p = 0.037). The EEG in VA-ECMO patients was less likely to have sleep architecture (3.0% vs. 28.6%, p = 0.044), while all other EEG findings were similar between the groups (Tables 3, 4).

**Table 3:** Comparison of baseline characteristics of patients who underwent VA-ECMO versus VV-ECMO A p-value <0.05 was considered statistically significant. ARDS, acute respiratory distress syndrome; EF, ejection fraction; VA, venoarterial; VV, venovenous

	VA-ECMO (n = 85)	VV-ECMO (n = 9)	p-value
Age, mean (SD), y	59.08 (12.20)	45.89 (16.26)	0.004
Indication for ECMO, No. (%)			
Respiratory failure	24 (28.2%)	7 (77.8%)	0.005
Cardiogenic shock or cardiac arrest	59 (69.4%)	1 (11.1%)	0.001
Septic shock	6 (7.1%)	2 (22.2%)	0.17
Cardiac surgery	13 (15.3%)	0 (0%)	0.35
Gender, No. (%)			0.067
Male	57 (67.1%)	3 (33.3%)	
Female	28 (32.9%)	6 (66.6%)	
Race, No. (%)			0.74
Caucasian	46 (59.0%)	6 (66.7%)	
Other	32 (41.0%)	3 (33.3%)	
Medical history, No. (%)			
Sepsis	38 (44.7%)	3 (33.3%)	0.73
ARDS	20 (23.5%)	9 (100%)	<0.001
Lung transplant	7 (8.2%)	2 (22.2%)	0.21
Heart failure	58 (69%)	0 (0%)	<0.001
Atrial fibrillation	38 (45.2%)	1 (11.1%)	0.07
Coronary artery disease	47 (55.3%)	1 (11.1%)	0.01
Myocardial infarction	17 (20%)	0 (0%)	0.36
Coronary artery bypass grafting	21 (25%)	0 (0%)	0.2
Use of mechanical circulatory support, No. (%)	43 (50.6%)	0 (0%)	0.003
The lowest recorded EF, No. (%)			0.003
<35%	44 (46.8%)	0 (0%)	
≥35%	41 (48.2%)	9 (100%)	
Wall motion abnormality, No. (%)	47 (55.3%)	0 (0%)	0.003
Left atrial enlargement, No. (%)	25 (29.4%)	1 (11.1%)	0.44
Life-threatening arrhythmia, No. (%)	33 (38.3%)	0 (0%)	0.024
Cannulation site, No. (%)			0.99
Central	16 (18.8%)	1 (11.1%)	
Peripheral	69 (81.2%)	8 (88.9%)	

**Table 4:** Comparison of outcomes for patients who underwent VA-ECMO versus VV-ECMO A p-value <0.05 was considered statistically significant. ^a ^Data missing for 24 patients ARDS, acute respiratory distress syndrome; ECMO, extracorporeal membrane oxygenation; EEG, electroencephalography; GPD, generalized periodic discharge; L MCA, left middle cerebral artery; LR, Lindegaard ratio; PDR, posterior dominant rhythm; PI, pulsatility index; R MCA, right middle cerebral artery; TCD, transcranial Doppler; VA, venoarterial; VV, venovenous

	VA-ECMO (n = 85)	VV-ECMO (n = 9)	p-value
Length of stay, median (IQR), d	22.0 (12, 39)	30 (20.5, 86)	0.104
ECMO duration, median (IQR), d	7 (4, 11.5)	8 (4.5, 15.5)	0.47
Outcome at time of discharge, No. (%)			0.3
Death	51 (62.2%)	4 (44.4%)	
Other (rehab, home, or transfer)	31 (37.8%)	5 (55.6%)	
Mortality after ECMO decannulation, No. (%)			
At 24 hours	35 (41.2%)	3 (33.3%)	0.74
At 30 days	48 (57.1%)	3 (33.3%)	0.29
At 365 days	54 (68.4%)	4 (44.4%)	0.26
Head CT findings, No. (%)			
Hypoxic-ischemic encephalopathy	3 (3.7%)	0 (0%)	0.99
Acute ischemic stroke	18 (22%)	0 (0%)	0.196
Intracranial hemorrhage	4 (4.9%)	0 (0%)	0.99
Subarachnoid hemorrhage	2 (2.5%)	0 (0%)	0.99
Subdural hemorrhage	1 (1.2%)	0 (0%)	0.99
TCD variables, median (IQR) (n = 75)^a^			
Average L MCA velocity	56.8 (44.76, 65.09)	78.7 (55.23, 95.45)	0.027
Average L ICA velocity	38.7 (31.35, 45.52)	40.4 (30.23, 49.55)	0.94
Average L LR	1.49 (1.15, 1.77)	1.57 (1.49, 2.52)	0.055
Average R MCA velocity	49.68 (42.15, 63.47)	75.03 (55.8, 88.1)	0.026
Average R ICA velocity	35.78 (29.13, 45.10)	37.7 (25.2, 45.8)	0.85
Average R LR	1.44 (1.19, 1.86)	2.18 (1.71, 3.35)	0.037
Average L MCA PI	0.96 (0.71, 1.37)	1.11 (1.03, 1.64)	0.052
Average R MCA PI	1.00 (0.73, 1.25)	1.16 (0.97, 1.36)	0.17
Average L MCA reactivity index	0.57 (0.49, 0.71)	0.64 (0.60, 0.74)	0.095
Average R MCA reactivity index	0.60 (0.49, 0.69)	0.65 (0.60, 0.70)	0.206
EEG findings, No. (%)			
Clinical seizure	1 (1.2%)	0 (0%)	0.99
Epileptiform discharges	2 (3.1%)	0 (0%)	0.99
Electrographic seizure	1 (1.5%)	0 (0%)	0.99
EEG variability	27 (43.5%)	5 (83.3%)	0.092
EEG reactivity	30 (47.6%)	2 (40%)	0.99
Focal asymmetry	6 (9.1%)	0 (0%)	0.99
PDR	7 (10.8%)	1 (14.3)	0.58
GPDs with triphasic morphology	11 (16.7%)	0 (0%)	0.59
Sharp waves	4 (6.1%)	2 (28.6%)	0.099
Periods of discontinuity	3 (4.5%)	0 (0%)	0.99
Sleep architecture	2 (3%)	2 (28.6%)	0.044

Twenty-three patients, all of whom had VA-ECMO (p = 0.09), had an adverse neurologic event: acute ischemic stroke (n = 18; 22%), intraparenchymal hemorrhage (n = 4, 4.9%), hypoxic-ischemic encephalopathy (n = 3, 3.7%), subarachnoid hemorrhage (n = 2, 2.5%), and subdural hemorrhage (n = 1, 1.2%). Patients with an adverse neurologic event were more likely to have received mechanical circulatory support (65.2% vs. 40.0%, p = 0.04), and they had lower average left ICA (32.35 vs. 40.4, p = 0.049) and right ICA velocities (33.3 vs. 37.0, p = 0.10).

## Discussion

Our study showed the feasibility and safety of MNM in ECMO patients. We found a higher rate of neurologic complications, specifically ischemic events, in patients undergoing VA-ECMO as opposed to VV-ECMO. We also found subtle differences in TCD and EEG characteristics between VA- and VV-ECMO patients and between survivors and non-survivors, suggesting that these characteristics are potential predictors of outcome and also hold prognostic value.

To our knowledge, ours is one of the largest cohorts of patients on ECMO to undergo protocolized MNM. Previously reported data are limited in scope and sample size [10]. Cho et al. reported an approach combining EEG, TCD, and somatosensory evoked potentials [8,11]. Their results suggested that MNM is safe and feasible for patients undergoing ECMO. In our patient cohort, MNM was completed in a timely manner for close to 75% of the patients, and all patients received at least one component of the monitoring protocol. No MNM-related adverse events were reported. Hence, our study further supports the feasibility and safety of MNM in patients on ECMO.

Shi et al. examined the usefulness of MNM in precisely identifying neurologic complications and its influence on long-term outcomes [12]. They showed that MNM improved precision in detecting neurologic injury and also improved long-term outcomes in ECMO survivors who had received MNM. In contrast, Ong et al. looked at a large cohort of ECMO patients and found an elevated incidence of neurologic complications in patients who underwent MNM [13]. Together, these results suggest that MNM allows early detection of neurologic complications but does not necessarily help clinicians prevent them. Nonetheless, early detection probably facilitates timely intervention and may improve outcomes [14]. Unfortunately, our patient cohort consisted only of patients who underwent the MNM protocol; hence, we are unable to explore differences between ECMO patients who did and did not undergo MNM.

It has been hypothesized that, compared with VV-ECMO, VA-ECMO is associated with higher rates of neurologic complications because blood is directly pumped into the arterial circulation and bypasses the filtration of thrombi that would occur in the lungs with VV-ECMO. However, a recent study of ECMO in both pediatric and adult patients found that the rates of neurologic complications were similar between VA- and VV-ECMO [3,15,16]. We had a total of 23 adverse neurologic events, all of which occurred in the VA-ECMO group and most of which were ischemic. Adverse neurologic event rates have been reported at 20% for pediatric patients [17] and at 1-33% for adults [18]. Hemorrhagic complications in adults are reportedly more common [19], but this was not seen in our patient cohort. This difference could have been the consequence of a revised anticoagulation protocol that was initiated at our institution before the start of our study [9].

Seizures are the least common neurologic complication of ECMO, being reported at a rate of 1-2% [20]. Among the patients who underwent EEG monitoring in our study, 1.8% had clinical seizures, which is similar to rates in prior reports. Lower seizure rates in ECMO-supported patients may be due to the rigorous sedation protocol that these patients undergo, which may have anticonvulsant effects.

The MCA velocities and LR did not appear to be significantly different between survivors and non-survivors. Left MCA PI and RI were not statistically different between groups, either, although the small p-value might indicate that using a larger sample size would have given the analyses enough power to identify a difference. There was, however, a statistically significant between-groups difference in right MCA PI and RI. Lower-than-normal PI values have been previously reported in ECMO patients [21] and have been attributed to depressed cardiac function [22,23]. Lower values have been associated with poor outcomes and have been predominantly studied in the context of brain death. Non-survivors had lower ejection fractions, and the lower PI and RI could possibly have been confounded by the underlying cardiac condition. Also, the differences in PI and RI were not apparent between patients who had an adverse neurologic event and patients who had no such events. Our results suggest that lower PI and RI are related to poor overall outcomes but are not necessarily correlated with adverse neurologic events. However, these conclusions are limited by the fact that our sample size was too small for us to perform a logistic regression that would produce meaningful results; thus, we could not control for the influence of other factors.

In comparing VA- and VV-ECMO, the primary differences were found in the indication for ECMO, medical history, and variables related to cardiac dysfunction. This is expected, given the different clinical scenarios in which each circuit is used. Below-normal MCA velocities have been reported in pediatric patients [24], but a difference in the velocities between VA- and VV-ECMO has not previously been reported [25]. We found higher MCA velocities and LR in patients undergoing VV-ECMO. The LR is the ratio of the MCA velocity to the ICA velocity and helps differentiate hyperemia from vasospasm, with higher values favoring vasospasm. This difference may be the result of intact native heart function and the pulsatility of blood flow through the cerebral vasculature in VV-ECMO patients. Another possible cause is disruption of cerebral autoregulation, or vasospasm. Cerebral autoregulation is known to be altered in patients undergoing ECMO [26], and the degree of altered cerebral autoregulation has been correlated with imaging findings and neurologic outcomes [27]. Despite the higher average MCA velocities and LR, none of the VV-ECMO patients in our study had an adverse neurologic event. The clinical implications of these findings need to be explored further.

There is little data regarding EEG findings in patients undergoing ECMO. Cho et al. described a neurophysiologic pattern of loss of reactivity with a relatively preserved background [8]. In our cohort, EEG variability and reactivity were lost in 66% of patients, although significant differences between groups in EEG patterns were not apparent other than differences in sleep architecture. Sleep architecture is part of normal EEG findings, and its absence may indicate structural pathology, global cerebral dysfunction due to the use of sedatives, or encephalopathies due to metabolic derangements. VV-ECMO patients were more likely to have preserved sleep architecture, possibly because these patients had lower rates of neurologic complications and hence an overall better-preserved neurologic status. In addition, differences in the amount of sedation can be related to loss of sleep architecture in VA-ECMO patients; however, the dose of sedative agents was not recorded and compared between the two groups.

Patients with poor outcomes had a longer duration of ECMO, which was probably due to difficulty in weaning and non-resolving cardiopulmonary dysfunction. These patients had shorter overall lengths of stay, probably as a consequence of withdrawal of care, which was often done because of a poor neurologic prognosis. Neurological prognostication in this patient cohort is extremely difficult and confounded by multiple variables, such as sedation. Some authors have suggested that the association between poor prognosis and poor outcomes is the result of a self-fulfilling prophecy: A patient who is expected to have a poor outcome undergoes withdrawal of care, which “fulfills” the expectation of a poor outcome [28,29]. Although this notion is not clearly supported by our findings, an additional benefit of multimodal neuromonitoring could be a more accurate determination of neurological prognosis, which could promote more informed decision-making about withdrawal of care.

Biomarkers such as neuron-specific enolase have recently emerged as valuable prognostic tools and have been shown to correlate with 28-day mortality and CT findings [30]. These biomarkers could be used in conjunction with MNM results to identify patients who are likely to have worse outcomes.

Limitations

One of the limitations of our study was that we did not have long-term or continuous TCD data. Using median values might have canceled out significant, short-lived changes. Furthermore, TCDs were performed for a short duration and hence provide only a snapshot representation of the cerebral blood flow; thus, they might not have captured the full variability of flow dynamics in the cerebral vasculature. Future studies might benefit from using continuous or longer-duration TCD monitoring.

We did not have access to the patient’s hemodynamic data, ECMO flows, dosages of vasoactive medications, PCO2, or hemoglobin levels at the time of the TCD readings. Observing the real-time relationships among these clinical data could have allowed more sophisticated correlations with the TCD data.

We also did not include a sedation regimen, the need for renal replacement therapy, and several other clinical parameters. Nevertheless, part of the focus of the project was to determine the utility of collecting the data to better define how to use MNM in the care of ECMO patients going forward.

Another major limitation of our study is that we did not have a comparison group of patients who did not undergo MNM. Hence, we are unable to explore the important question of whether MNM has value in preventing neurologic injury or improving outcomes.

## Conclusions

Our study showed the feasibility, practicality, and safety of MNM in patients undergoing ECMO, and it identified EEG and TCD characteristics that might aid in the early detection of neurologic deterioration. Hence, MNM may have a role not just in monitoring patients undergoing ECMO but also in prognostication and in aiding clinical decision-making. Data from MNM could be utilized to individualize clinical parameters such as blood pressure goals, transfusion triggers, and anticoagulation targets. A wider adoption of MNM and further research are needed.
